# Predictors of physical activity and smoking among German teachers during the SARS-CoV-2 pandemic

**DOI:** 10.1093/eurpub/ckac130.137

**Published:** 2022-10-25

**Authors:** V Eggert, C Koestner, T Beutel, K Kalo, T Dicks, C Zaehme, S Letzel, P Dietz

**Affiliations:** 1 Research Group Public Health and Social Medicine, University Medical Center of the University of Mainz, Mainz, Germany; 2 Institute for Teachers’ Health, University Medical Center of the University of Mainz, Mainz, Germany; 3 Department of Sports Medicine, Disease Prevention and Rehabilitation, Johannes Gutenberg University Mainz, Mainz, Germany

## Abstract

**Background:**

The SARS-CoV-2 pandemic has influenced the social and health-related behavior due to significant changes and constraints in the professional and private life. Especially in the school context, there were considerable changes, which may have promoted positive and negative health behaviors. Therefore, the aim of our study was to identify the predictors of physical activity and smoking of teachers during the SARS-CoV-2 pandemic.

**Methods:**

In March 2021, a nation-wide online survey was conducted among teachers in Germany. A total number of 31,089 participants entered analysis. Data on smoking and physical activity as well as sociodemographic, workplace-related, psychological, SARS-CoV-2-related, and health-related items were collected using established instruments and if necessary self-developed items. Two binary logistic regressions with block wise inclusion of the different variable groups were performed to predict physical activity and smoking.

**Results:**

Among all surveyed teachers, 70.1% did not comply with the WHO recommendation of being physically active for at least 150 minutes per week and 13.9% reported to smoke. The regression analyses revealed significant predictors for physical activity (e.g., time requirement) and smoking (e.g., work schedule).

**Conclusions:**

Recommendations to improve teachers’ health can be derived from the predictors for physical activity and smoking that were identified in our study. Given the alarming result that more than two thirds of the teachers did not comply with the WHO recommendations, a special focus should be placed on improving physical activity, as this is a crucial factor for somatic and mental health.

**Key messages:**

- Teachers in Germany showed alarmingly low compliance regarding the WHO recommendations on physical activity during the pandemic.
- To improve teachers’ health, it is important to take into account significant predictors of health behavior when planning interventions.

